# The prevalence of severe fatigue in rheumatic diseases: an international study

**DOI:** 10.1007/s10067-015-3035-6

**Published:** 2015-08-15

**Authors:** Cécile L. Overman, Marianne B. Kool, José A. P. Da Silva, Rinie Geenen

**Affiliations:** Department of Clinical and Health Psychology, Utrecht University, PO Box 80.140, 3508 TC Utrecht, The Netherlands; Department of Rheumatology, Hospitais da Universidade de Coimbra, 3000-075 Coimbra, Portugal; Department of Rheumatology and Clinical Immunology, University Medical Center Utrecht, PO Box 85.500, 3508 GA Utrecht, The Netherlands

**Keywords:** Fatigue, Fibromyalgia, Osteoarthritis, Rheumatic diseases, Rheumatoid arthritis, Vitality

## Abstract

Fatigue is a common, disabling, and difficult-to-manage problem in rheumatic diseases. Prevalence estimates of fatigue within rheumatic diseases vary considerably. Data on the prevalence of severe fatigue across multiple rheumatic diseases using a similar instrument is missing. Our aim was to provide an overview of the prevalence of severe fatigue across a broad range of rheumatic diseases and to examine its association with clinical and demographic variables. Online questionnaires were filled out by an international sample of 6120 patients (88 % female, mean age 47) encompassing 30 different rheumatic diseases. Fatigue was measured with the RAND(SF)-36 Vitality scale. A score of ≤35 was taken as representing severe fatigue (90 % sensitivity and 81 % specificity for chronic fatigue syndrome). Severe fatigue was present in 41 to 57 % of patients with a single inflammatory rheumatic disease such as rheumatoid arthritis, systemic lupus erythematosus, ankylosing spondylitis, Sjögren’s syndrome, psoriatic arthritis, and scleroderma. Severe fatigue was least prevalent in patients with osteoarthritis (35 %) and most prevalent in patients with fibromyalgia (82 %). In logistic regression analysis, severe fatigue was associated with having fibromyalgia, having multiple rheumatic diseases without fibromyalgia, younger age, lower education, and language (French: highest prevalence; Dutch: lowest prevalence). In conclusion, one out of every two patients with a rheumatic disease is severely fatigued. As severe fatigue is detrimental to the patient, the near environment, and society at large, unraveling the underlying mechanisms of fatigue and developing optimal treatment should be top priorities in rheumatologic research and practice.

## Introduction

Fatigue is a common problem in patients with a rheumatic disease. It can be as disabling as pain, is difficult to manage, and has a substantial impact on quality of life [1–10]. In research and clinical practice, multiple instruments have been used to assess fatigue, representing disease-specific versus generic as well as unidimensional versus multidimensional definitions of fatigue [4, 11, 12]. As a consequence, it is difficult to determine how widespread severe fatigue really is among patients with rheumatic diseases. Indeed, prevalence estimates of fatigue vary considerably within rheumatic diseases [4], and the evaluation of the prevalence of severe fatigue with a uniform measure across different rheumatic diseases has been limited to comparing just a few rheumatic diseases at a time [3, 13]. In this study, we chose a common generic (not disease-specific), unidimensional specification of fatigue to be able to estimate the prevalence of a severe level of fatigue across multiple rheumatic diseases.

Fatigue has been indicated to be more prevalent in patients with more than one rheumatic disease [3], and there are some indications for a higher prevalence in women [2, 5] and in people with a lower social economic status [14]. So far, no significant associations have been reported between fatigue and other demographics such as age and ethnicity [2]. Considering that fatigue is a core symptom of fibromyalgia [15], a high prevalence of severe fatigue in patients with fibromyalgia is an obvious expectation. However, evidence that severe fatigue is higher in patients with a rheumatic disease and comorbid fibromyalgia is mixed [16–18].

The main objective of the current study was to provide an overview of the prevalence of severe fatigue across a broad range of rheumatic diseases in a single large international sample of patients using a uniform measure of fatigue and a stringent cutoff criterion indicating a level of fatigue that is comparable with fatigue in patients with chronic fatigue syndrome. A secondary objective was to examine in which subgroups of patients severe fatigue is more prevalent; subgroups were defined by the presence of (comorbid) fibromyalgia or multiple rheumatic diseases not including fibromyalgia, disease duration, and demographic variables.

## Methods

### Participants

The study population comprised 6120 patients with rheumatic diseases from different countries who participated between November 2009 and September 2011 in an online study that examined invalidation (i.e., patients’ perception of responses of others that are denying, lecturing, not supporting, and not acknowledging the condition of the patient [19]). Allied researchers from several European countries and the USA asked patient associations for rheumatic diseases in Europe and North and South America to put a recruitment notice with a hyperlink to the online questionnaire on their websites. The text of the recruitment notice was similar across languages and included information about the aim and content of the study, inclusion criteria, and duration of participation (about 20 min). Based on this information, people could decide to participate while being able to stop at any time if they desired. Inclusion criteria were a self-reported diagnosis of at least one rheumatic disease, being 18 years or older, and speaking one of the languages in which the online questionnaires were provided (i.e., Dutch, English, French, German, Portuguese, or Spanish). The study was conducted according to the principles of the Declaration of Helsinki [20] and approved by the medical ethical review board of the University Medical Center Utrecht. Further details of the study have been described [19].

### Materials

In the online study, participants were asked which rheumatic diseases they had, the disease duration, and demographic characteristics. Several questionnaires were included. Relevant for the current study was that fatigue was measured with the Vitality scale of the RAND version of the SF 36-item Health Survey, the RAND(SF)-36 [21–23]. For the Vitality scale, both the content and scoring is equal across versions. The reliability and validity of this questionnaire have been tested for all languages included in this study and are satisfactory in both the general population and in patient samples [24–30]. The Vitality scale assesses a general level of fatigue in the last 4 weeks using four items (i.e., Did you *feel full of life*/*have a lot of energy*/*feel worn out*/*feel tired*) scored on a 6-point Likert scale (*all*/*most*/*a good bit*/*some*/*a little*/*none* of the time). The final score range is 0–100 with lower scores representing more fatigue. In our study, scores of 35 or lower were considered to indicate severe fatigue. This cutoff score is similar to the 10th percentile of the general population [31]. To identify patients with chronic fatigue syndrome, this cutoff score was found to have 90 % sensitivity (i.e., 90 % of people with chronic fatigue syndrome according to established classification criteria were correctly identified as having chronic fatigue syndrome using this cutoff score) and 81 % specificity (i.e., 81 % of the people not having chronic fatigue syndrome according to established classification criteria were correctly identified as not having chronic fatigue syndrome using this cutoff score) [32].

### Statistical analysis

In descriptive analyses, the cutoff score of ≤35 was used to evaluate the number of patients with severe fatigue for groups of patients with a single rheumatic disease or multiple rheumatic diseases. Single rheumatic diseases were represented by a minimum of 75 patients. Single rheumatic diseases represented by less than 75 patients were included in analyses as one combined group, “a single other rheumatic disease.”

Logistic regression analysis examined the prevalence of severe fatigue in specific groups. Standard descriptive variables gender, age, number of years of education (≤14 and >14), marital status (single or divorced/widowed, with “being in a relationship” as the reference category), and language (English, French, German, Portuguese, and Spanish, with Dutch as the reference category) were available in the dataset and included in the analysis. Of disease-related variables, disease duration and the presence of (comorbid) fibromyalgia and multiple rheumatic diseases (not including fibromyalgia) were included; (comorbid) fibromyalgia, which was our largest subgroup and has fatigue as part of the preliminary diagnostic criteria [15], was the only rheumatic disease included. In case of multiple rheumatic diseases, the longest disease duration was selected. The variable disease duration was log transformed to correct the positively skewed score distribution.

For the logistic regression analysis, regression coefficients (*B*), standard errors (SE), Wald statistics, logistic pseudo partial correlations (*r*, i.e., the explanatory value attributable to a single independent variable after adjusting for all other independent variables), odds ratios, and goodness of fit of the whole model (*Nagelkerke*’*s R*^2^) are reported. Significance levels were set at *p* < 0.05. Data were analyzed using SPSS 20.

## Results

### Characteristics of the study population

Table 1 shows the characteristics of the 6120 patients with rheumatic diseases. Almost half of the patients reported a diagnosis of fibromyalgia (49 %). The most commonly reported combinations of rheumatic diseases were fibromyalgia and osteoarthritis (*n* = 511), fibromyalgia and osteoarthritis and another rheumatic disease (*n* = 203), osteoarthritis and other rheumatic diseases not being fibromyalgia (*n* = 228, of which 102 had rheumatoid arthritis), and rheumatoid arthritis and other rheumatic diseases not being osteoarthritis or fibromyalgia (*n* = 181). In total, this study covered 30 rheumatic diseases.

**Table 1:** Characteristics of the 6120 patients with rheumatic diseases

Female sex, *n* (%)	5391	(88)
Age, mean ± SD	47	±12
Men	50	±12
Women	46	±12
Years of education, *n* (%)		
≤14 years	2673	(44)
>14 years	2959	(48)
Unknown	488	(8)
Marital status, *n* (%)		
Single	963	(16)
Married or in a steady relationship	4480	(73)
Separated or widowed	662	(11)
Unknown	15	(0)
Language, *n* (%)		
Dutch	1871	(31)
English	739	(12)
French	787	(13)
German	560	(9)
Portuguese	725	(12)
Spanish	1438	(23)
Disease duration, median, interquartile range	5	2–11
Rheumatic disease, *n* (%)^a^		
Fibromyalgia	2993	(49)
Osteoarthritis	1249	(20)
Rheumatoid arthritis	1054	(17)
Systemic lupus erythematosus	804	(13)
Ankylosing spondylitis/Bechterew’s disease	621	(10)
Sjögren’s syndrome	567	(9)
Psoriatic arthritis	240	(4)
Scleroderma	147	(2)
Polymyalgia rheumatica	93	(2)
Ehlers-Danlos syndrome or hypermobility syndrome	85	(1)
Juvenile idiopathic arthritis	81	(1)
Gout or pseudogout	62	(1)
Mixed connective tissue disease	56	(1)
Tietze’s syndrome/costochondritis	54	(1)
Another rheumatic disease^b^	149	(2)

^a^Due to patients with more than one rheumatic disease, the sum of percentages mentioned per rheumatic disease exceeds 100 %

^b^The most mentioned diseases in the category “another rheumatic disease” are osteoporosis (*n* = 22), Behçet’s disease (*n* = 21), Still’s disease (*n* = 21), sarcoidosis (*n* = 18), undifferentiated spondyloarthropathy (*n* = 15), and dermatomyositis (*n* = 11)

Participants resided in various countries around the world. The most common countries of residence per language were as follows: Dutch speakers lived mostly in the Netherlands (*n* = 1613) and Belgium (*n* = 236); English speakers in the UK (*n* = 450), the USA (*n* = 104), and Canada (*n* = 78); German speakers in Germany (*n* = 520); French speakers in France (*n* = 680) and Belgium (*n* = 62); Spanish speakers in Spain (*n* = 811), Argentina (*n* = 266), Mexico (*n* = 132), and Chile (*n* = 54); and Portuguese speakers in Portugal (*n* = 687).

### Prevalence of severe fatigue

**Fig. 1:**
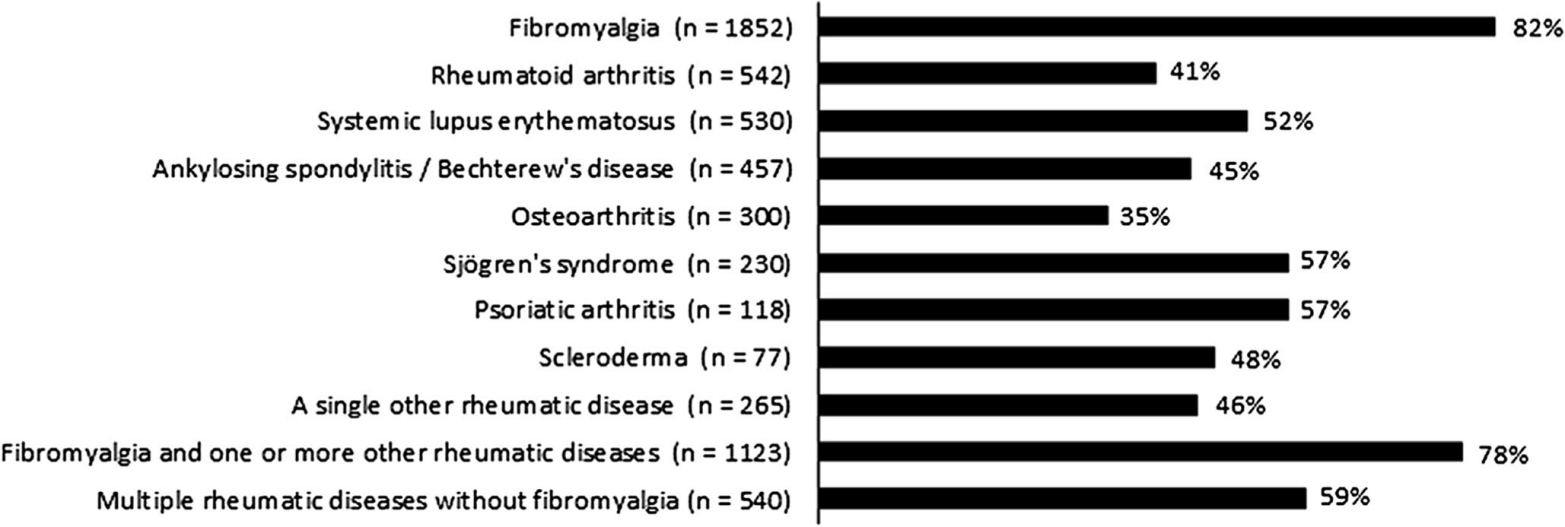
Prevalence of severe fatigue [RAND(SF)-Vitality score ≤35] in patients with rheumatic diseases. Of the 6120 patients, 6034 had a SF-Vitality score; the number of patients with a missing score ranged per rheumatic disease group from 3 to 16. “A single other rheumatic disease” included all diagnoses which did not reach the minimum of 75 patients to represent a specific rheumatic population. Patients with multiple rheumatic diseases were divided into a group with fibromyalgia and a group without fibromyalgia as one of the diagnoses

The prevalence of severe fatigue is shown in Fig. 1 for patients with a single rheumatic disease and patients with multiple rheumatic diseases with or without fibromyalgia. Severe fatigue was present in 65 % of all patients, with percentages from 41 to 57 % in patients with a single inflammatory rheumatic disease, around 80 % in patients with fibromyalgia, and 35 % in patients with osteoarthritis.

### Associations with severe fatigue

The logistic regression analysis (Table 2) showed that having fibromyalgia, having multiple rheumatic diseases without fibromyalgia, and being a member of the sample that did not speak Dutch increased patients’ chance of being severely fatigued while having a longer disease duration, being older, and having more years of education decreased patients’ chance. Gender and marital status were not significantly associated with severe fatigue (*p*s ≥ 0.19). According to the partial correlation adjusted for the other variables, having fibromyalgia had the strongest association with severe fatigue of all included independent variables (*r* = 0.23). For patients with fibromyalgia, it was four times more likely to be severely fatigued than for patients without fibromyalgia. Language was another variable with a strong association with severe fatigue; most notably, for French speakers, the chance of being severely fatigued was almost six times higher than for Dutch speakers. The logistic analysis showed that, overall, severe fatigue was less likely in patients who spoke Dutch than in patients with another language. Supplementary analyses showed that 64 % of Dutch-speaking patients with fibromyalgia were severely fatigued versus 85 to 91 % of patients with fibromyalgia in populations with another language. The fit of the total model (Nagelkerke’s *R*^2^) was 0.23, leaving more than 75 % of severe fatigue unexplained.

**Table 2:** Logistic regression model examining associations with severe fatigue (RAND(SF)-Vitality ≤35)

	*B* (SE)	Wald statistic	*r* ^a^	Odds ratio
Fibromyalgia	1.35 (0.07)	368.16***	0.23	3.86
Multiple rheumatic diseases without FM^b^	0.49 (0.11)	20.95***	0.05	1.63
Disease duration^c^	−0.27 (0.08)	11.60**	0.04	0.76
Gender^d^	−0.12 (0.10)	1.69	0.00	0.88
Age	−0.01 (0.003)	11.15**	0.04	0.99
Years of education^e^	−0.30 (0.07)	19.87***	0.05	0.74
Marital status^f^				
Single	−0.08 (0.09)	0.93	0.00	0.92
Separated/widowed	0.01 (0.11)	0.03	0.00	1.01
Language^g^				
English	1.12 (0.12)	88.44***	0.11	3.08
French	1.81 (0.13)	208.62***	0.17	6.11
German	0.94 (0.12)	65.64***	0.09	2.57
Portuguese	0.34 (0.11)	10.46**	0.03	1.41
Spanish	0.74 (0.08)	78.35***	0.10	2.09

* = *p* < 0.05; ** = *p* < 0.01; *** = *p* < 0.001; “Variance” explained by the total model, Nagelkerke’s *R* ^2^ = 0.23

^a^ *r* is the logistic pseudo partial correlation, i.e., the explanatory value attributable to a single independent variable after taking into account all other independent variables

^b^Having multiple rheumatic diseases without fibromyalgia (FM): yes = 1, no = 0

^c^Disease duration is log transformed

^d^Gender: male = 1 and female = 0

^e^Years of education: >14 years = 1, ≤14 years = 0

^f^Marital status: two dummy variables with “in a relationship” as reference category

^g^Language: five dummy variables with Dutch as reference category

## Discussion

This study in 30 rheumatic diseases shows that severe fatigue is a widespread and highly prevalent problem across rheumatic diseases. Overall, one out of every two patients was severely fatigued. Severe fatigue was least common in patients with osteoarthritis (35 %) and most common in patients with (comorbid) fibromyalgia (around 80 %). Patients’ odds of being severely fatigued were higher when having fibromyalgia, multiple rheumatic diseases without fibromyalgia, a shorter disease duration, a younger age, and less years of education; also language was related to fatigue.

Thorough research into the prevalence of severe fatigue in rheumatic diseases would demand random sampling, certification of the rheumatic disease by a medical specialist, and identifying chronic fatigue using classification criteria next to self-report scores as has been done in chronic fatigue syndrome [33]. This type of prevalence studies have not been done yet. Previous studies examining fatigue across rheumatic diseases all had their limitations. One study examined three rheumatic diseases and, like most other studies [5–7, 10], used a sample of patients recruited in one rheumatology clinic [13]. Another used a sample of the general population in which the presence of a rheumatic disease was determined by self-reported diagnosis and did not present a cutoff for fatigue or prevalence per rheumatic disease [3]. Overall measures to assess fatigue varied across rheumatic diseases and in definitions of fatigue severity [5–7, 9, 10] which impeded insight into the prevalence of severe fatigue in distinct rheumatic diseases. A strength of our study is that a uniform measure and cutoff were used to estimate the prevalence in distinct rheumatic diseases. In spite of the diversity of assessment and sampling methods in all studies thus far, our prevalence estimates are in agreement with previous studies examining the prevalence of severe fatigue [7, 13, 34] and lower than studies examining prevalence of less severe levels of fatigue in rheumatic diseases [6, 9, 35].

Although having (comorbid) fibromyalgia increased patients’ odds of being severely fatigued, this study also clearly showed that severe fatigue is by no means exclusive to patients with (comorbid) fibromyalgia; around 50 % of patients with other rheumatic diseases are also severely fatigued. Indeed, fatigue has been recognized in recent years as a core symptom and outcome measure not only in fibromyalgia but also in rheumatoid arthritis, psoriatic arthritis, and ankylosing spondylitis [2, 5, 9, 15]. Patient-focused group discussions indicated that fatigue is overwhelming and different from normal tiredness, that it permeates every sphere of life, and that the possibilities of self-management are variable and professional support is rare [2, 36]. The current study showed high prevalence of severe fatigue across 30 rheumatic diseases and different cultural backgrounds. This suggests that fatigue should be considered a core symptom and outcome measure in clinical trials and clinical practice for all rheumatic diseases, without exception. Moreover, the finding emphasizes that the development and evaluation of adequate management and treatment of fatigue in rheumatic diseases is of utmost importance.

The pathology of fatigue is largely unknown. The high prevalence of fatigue in rheumatic diseases suggests that the inflammatory process is a precipitating and possible maintaining factor of fatigue. This interpretation is somewhat supported by the observation of a lower prevalence of fatigue among patients with osteoarthritis, but it is contradicted by the high prevalence being observed in fibromyalgia. Moreover, associations of clinical and laboratory variables with fatigue are mostly low or absent [1, 2]. With current knowledge, “acute” fatigue as a result of a disease flare-up is probably best targeted by pharmacological interventions, while behavioral means such as life-style adjustment, cognitive behavioral therapy, (graded) physical exercise training, and sleep hygiene interventions should be considered in the treatment of chronic fatigue [1, 37, 38].

The study being international increased the probability that the findings are generalizable to different countries and cultures, but it also indicated some differences in the prevalence of severe fatigue between people with a different language. The presence of severe fatigue varied substantially across languages with French-speaking patients most often reporting severe fatigue and Dutch-speaking patients least often. Similar findings have been reported for functioning and wellbeing, as measured with the RAND(SF)-36, both between countries and between language regions, with Dutch speakers having more favorable scores than people with another language and French speakers scoring relatively poor [39, 40]. These differences do not seem to be due to measurement invariance [39, 41] but have been attributed to differences in access to and quality of national health systems [42], differences in social and economic opportunities within countries [43], and culture [44].

The current study has some limitations. It was based on self-reported diagnoses of rheumatic diseases without certification by a medical specialist, which may have led to the incorrect reporting of rheumatic diseases. Moreover, the recruitment through the Internet may have led to a lower representation of the older patient population and patients with a low social economic status, and it surely led to an overrepresentation of some patient groups (e.g., those with larger or more active patient associations). Furthermore, partly due to the overrepresentation of some patient groups such as fibromyalgia which are mostly female, men are underrepresented in this study. Finally, predominantly Western European countries participated in this study. Thus, our results might not be a fair reflection of the prevalence of severe fatigue in older, male patients and patients from other than Western European countries.

The use of a generic questionnaire to measure fatigue enabled us to measure the severity of fatigue in multiple diseases, and using a unidimensional measure may perhaps have reduced confounding by other states such as depressed mood that may differ among diseases. In rheumatoid arthritis, fatigue has been defined as multidimensional in nature, including physical, emotional, and cognitive aspects of fatigue and the daily impact of living with fatigue [45], a definition which can be useful in clinical assessment. As our goal was to give an overview of the prevalence of severe fatigue, not its impact, across a broad range of rheumatic diseases, we chose the Vitality scale of the RAND(SF)-36 which also had the advantage that the results are directly comparable to the level of fatigue in the general population.

A strength of the study is that a stringent uniform cutoff score was used, which has been shown sensitive to identify patients with chronic fatigue syndrome in the general population [32]. However, to assure that this cutoff indeed measures a level of severe fatigue comparable to chronic fatigue syndrome, future studies should examine if it is sensitive to identify severe fatigue using classification criteria other than self-report only [33] in patients with rheumatic diseases.

This study is the first to provide an overview of the presence of severe fatigue across 30 rheumatic diseases using a large international dataset, a uniform way of recruitment, a uniform measure to assess fatigue, and a verified [31, 32] cutoff score for severe fatigue. It showed that more than 50 % of all patients with a rheumatic disease are severely fatigued. Severe fatigue can have detrimental effects for the patient, the near environment, and society at large. A better understanding of fatigue is crucial. In rheumatology, unraveling the underlying mechanisms of fatigue and developing optimal treatments should be top priorities in research and clinical practice.
